# Radiation-related thyroid autoimmunity and dysfunction

**DOI:** 10.1093/jrr/rrx054

**Published:** 2017-10-24

**Authors:** Yuji Nagayama

**Affiliations:** Department of Molecular Medicine, Atomic Bomb Disease Institute, Nagasaki University, 1-12-4 Sakamoto, Nagasaki 852-8523, Japan

**Keywords:** thyroid, autoimmunity, hypothyroidism, hyperthyroidism, radiation

## Abstract

The thyroid gland is vulnerable not only to external radiation but also to internal radiation, because the thyroid cells can incorporate radioactive iodine when synthesizing thyroid hormones. Since radiation-induction of thyroid neoplasia, including thyroid cancer, is well recognized, the data on radiation-related thyroid autoimmunity and dysfunction are summarized and reviewed. High-dose irradiation, irrespective of being external or internal, is strongly associated with a risk of hypothyroidism (with the prevalence ranging from 2.4% to 31%) and of Graves’ hyperthyroidism (with the prevalence being up to 5%). It is easy to understand that high-dose irradiation induces hypothyroidism with some frequency, because high-dose irradiation destroys the thyroid gland. On the other hand, the basis for development of hyperthyroidism is mechanistically unclear, and it is merely speculative that autoantigens may be released from damaged thyroid glands and recognized by the immune system, leading to the development of anti-thyrotropin receptor antibodies and Graves’ hyperthyroidism in subjects who are immunologically predisposed to this ailment. In contrast, the data on moderate to low-dose irradiation on thyroid autoimmunity and dysfunction are inconsistent. Although it is difficult to draw a definitive conclusion, some data may suggest a transient effect of moderate- to low-dose irradiation on hypothyroidism and autoimmune thyroiditis, implying that the effect, if it exists, is reversible. Finally, no report has shown a statistically significant increase in the prevalence of moderate- to low-dose irradiation–induced Graves’ hyperthyroidism.

## INTRODUCTION

The thyroid gland is the largest classical endocrine organ (typically 15–20 g) and is located in the anterior neck. It incorporates and utilizes iodine to synthesize thyroid hormones, thyroxine (T_4_) and triiodothyronine (T_3_), which regulate growth, metabolism and other important physiological processes of the human body [1].

The thyroid gland is well known to be vulnerable to radiation. Not only thyroid cancers but also non-malignant thyroid diseases, including benign thyroid nodules, autoimmune thyroid diseases (such as Graves’ disease and Hashimoto’s thyroiditis) and also non-autoimmune (or destructive) hypothyroidism have been reported to be induced by a wide dose range of radiation. In this review, the data accumulated so far regarding the effect of various types and various doses of radiation on thyroid function and thyroid autoimmunity are summarized. Thyroid tumors are outside the scope of this review. The papers cited here were chosen from the reference lists obtained by searching the electronic databases of PubMed with search terms of ‘radiation’ AND ‘thyroid dysfunction’, ‘radiation’ AND ‘thyroid autoimmunity’, and ‘atomic bomb’ AND ‘thyroid’ for 30 years from 1987 to 2017, as well as from the reference lists of the articles obtained as above.

## THE EFFECT OF HIGH-DOSE RADIATION

High-dose irradiation has been reported to induce a variety of thyroid diseases, including thyroid dysfunction (hypothyroidism and hyperthyroidism) and thyroid neoplasia. It is easy to understand that high-dose irradiation (either internal or external) destroys the thyroid gland, leading to destructive hypothyroidism. Indeed, primary hypothyroidism is the most common consequence of high-dose irradiation. However, curiously, small percentages of patients with thyroid autonomy or non-thyroidal malignant diseases treated with high-dose internal or external irradiation, respectively, have been reported as developing Graves’ hyperthyroidism or autoimmune thyroiditis.

### External irradiation

High-dose external irradiation to the thyroid was performed in patients with non-thyroidal malignant diseases [such as Hodgkin disease (HD), acute lymphoblastic leukemia (ALL), etc.] who received irradiation to the neck region or as total-body irradiation (TBI) (Table 1).

**Table 1.** The incidence of thyroid dysfunction and autoimmune diseases in the subjects who received high-dose external irradiation

Authors [ref.]	Radiation doses [median, (range) Gy]	# pts	Diagnosis	Ages at diagnosis or initial treatment [median, (range)]	Hypo-thyroidism [%, (#)]	Hyper-thyroidism [%, (#)]	Thyroiditis [%, (#)]	Silent thyroiditis [%, (#)]	Latent periods (median (range) years)
Fleming [2]	(18–60)	298	HD, ALL, *etc*	(1.5–20)	8.7 (26)	0.3 (1)	0.7 (2)	–	hypo; 7 (1–16)
									thyroiditis; (1–7)
Hancock [3]	(15–44)	1677	HD	28 (2–82)	31 (512)	1.9 (32)	0.2 (4)	0.4 (6)	hypo; 4 (0.2–23.7)
									hyper; 4.9 (0.1–17.6)
									silent thyroiditis; (0.8–15)
Sklar [4]	35 (0.37–55)	1791	HD	14 (2–20)	25 (456)	4.6 (82)	–	–	hypo; 7 (0–27)
									hyper: 8 (0–22)
Khoo [5]	39.8 (32–65)	320	HD	30 (7–79)	35 (112)	4 (13)	–	–	9 (1–23)^a^
Schmiegelow [6]	51 (31–57)	71	brain tumors	8.4 (0.8–14.9)	24 (17)	0 (0)	–	–	12 (2–28)
Chow [7]	(<15–30)	2358	ALL	4 (0–20)	2.4 (56)	1.0 (23)	–	–	(15)
Thomas [8]	(10–13.5)	186	ALL, AML, *etc*	(>15)	6.5 (12)	1.5 (1)	3 (6)	–	hypo; 2.5 (1–8)
									thyroiditis; 1 (0.25–2)
									Graves; (1)

^a^follow-up periods.

In the report from St Jude Children’s Hospital [2], of 298 patients with malignant diseases (including HD, ALL, etc.), 26 developed subclinical (17/26) or overt (9/26) hypothyroidism (8.7%), and 1 (0.3%) developed hyperthyroidism. The ages at the time of the initial treatment in those who developed hypothyroidism were 1.5 to 11 years old (median, 10 years old), the radiation doses ranged from 24 to 60 Gy (median, 38 Gy), and the latent periods from the initiation of treatment to hypothyroidism were 1 to 16 years (median, 7 years). No information was available for a patient who developed hyperthyroidism. Thyroiditis developed in 2 patients (0.7%) following 18 and 20 Gy irradiation at the ages of 17 and 8 years with latent periods of 1 and 7 years, respectively, although the definition of thyroiditis was not clarified. They also summarized the papers published in the 1970s and early 1980s, which showed the incidences of subclinical and overt hypothyroidism reached >50% and ~25%, respectively, over longer follow-up periods.

In a study of patients with HD [3] who received 15 to 44 Gy (mainly 15 to 34 years old), 31% (512/1677) developed hypothyroidism, with a median time from the initiation of therapy to occurrence of hypothyroidism being 4.0 years (range, 0.2–23.7 years). The risk of hypothyroidism was dependent on radiation dose; the incidence of hypothyroidism was 44% in patients who received >30 Gy, 27% in those who received 7.5–30 Gy and 2% in those who received 0 Gy for 20 years. On the other hand, the incidence of hyperthyroidism was 1.9% (32/1677), with a median time to occurrence of 4.9 years (range, 0.1–17.6 years). The risk of developing hyperthyroidism was 3% in patients who received >30 Gy, 1% in those who received 7.5–30 Gy and 2% in those who received 0 Gy.

In the other study of HD patients [4], they received a median dose of radiation to the thyroid of 35 Gy (range, 0.37–55 Gy), and the incidence of hypothyroidism following radiotherapy was 25% (456 cases; ~17-fold greater than controls) during a median follow-up of 16 years. The risk of developing hypothyroidism was greatest in the first 5 years, but cumulatively 30% and 50% of patients who received 35–45 Gy and >45 Gy, respectively, developed hypothyroidism by 20 years after diagnosis of HD. The risk factors included increasing dose of radiation, female sex and older age at diagnosis. In addition, 5% of patients (82 cases; 8-fold greater than controls) developed hyperthyroidism, with the mean time between diagnosis of HD and the development of hyperthyroidism being 8 years (range, 0–22 years); a radiation dose of >35 Gy to the thyroid and <3 years since diagnosis were risk predictors.

Khoo *et al.* also reported the occurrence of thyroid autoimmunity and dysfunction in patients with HD following radiation therapy [5]; in 320 patients who received a median thyroid dose of 39.8 Gy (range, 32–65 Gy), 35% and 4% developed hypo- and hyperthyroidism, respectively, during the median follow-up period of 9 years (range, 1–23 years).

In a childhood (<15 years old) brain tumor study [6], 24% (17/71) of patients who received 24–57 Gy cranial/craniospinal irradiation developed hypothyroidism during the 2–28 year follow-up periods (median, 12 years). Hyperthyroidism was not observed in this study.

In ALL patients in the Childhood Cancer Survivor Study [7], ~2.4% (56/2326) of those who received cranial/spinal radiotherapy developed primary or central (due to irradiation to the pituitary gland) hypothyroidism by 15 years after diagnosis. Those who received cranial and spinal radiotherapy of >20 Gy were at the highest risk of hypothyroidism. Overall, 1% (23/2326) also developed hyperthyroidism in the 15 years following diagnosis. Those who received thyroid doses of >15 Gy had an increased risk of hyperthyroidism.

In the case of TBI, a retrospective study on patients with various hematological diseases has been reported [8]. In 186 patients who received a single TBI dose of 10 Gy or fractionated TBI of 12–13.5 Gy at ages of >15 years old, 6.5% (12/186) developed hypothyroidism, 3% (6/186) developed thyroiditis and 1.5% (1/186) developed Graves’ hyperthyroidism over a median follow-up period of 49 months (range, 12–136 months). Anti-thyroglobulin autoantibodies, as well as thyroid hormones, were measured to define thyroid abnormalities, but a detailed definition of thyroiditis was not provided.

### Internal irradiation

Radioactive iodine ^131^I has long been used as a treatment modality for Graves’ hyperthyroidism and other autonomous thyroid conditions such as toxic adenoma/multinodular goiter. A thyroid dose of ^131^I is typically 30–80 Gy [9]. ^131^I incorporated into the thyroid gland causes ionizing damage to the thyroid follicular epithelial cells, leading to gland destruction. The ideal goal of radioactive iodine therapy is to render the patients euthyroid, but the reality is to ablate the thyroid gland and to make the patients hypothyroid.

As an example, Metso *et al.* reported that the cumulative incidences of hypothyroidism in Graves’ disease and toxic multinodular goiter treated with ^131^I was 24% and 4% at 1 year after treatment, 59% and 15% at 10 years, and 82% and 32% at 25 years, respectively [10]. Similarly a high incidence of hypothyroidism after long-term follow-up was also noted by others; 18% at 5 years and 42% at 20 years [11], and 6% in 1 year and 72% at 20 years [12].

As another adverse effect of internal irradiation with radioiodine, radiation thyroiditis with transient thyrotoxicosis without hyperthyroidism can occur within 1 month after administration of ^131^I, which is attributed to radiation-induced acute destruction of the thyroid gland. However, paradoxically, radioiodine therapy induction of Graves’ hyperthyroidism has also been infrequently observed in the patients with toxic adenoma/multinodular goiter (Table 2).

**Table 2.** The incidence of Graves’-like hyperthyroidism in subjects who received high-dose internal irradiation

Authors [ref.]	Radiation dose [median, (range)]	Diagnosis	#pts	Hyper (%, #)	Latent period (range, months)
Nygaard [13]	370 (259–740) MBq	non-toxic goiter	191	5 (9)	3
Nygaard [14]	(<740 MBq, 1–5x)	toxic goiter	149	4 (5)	3–6
Dunkelman [15]	(74–1560 MBq, 150–400 Gy)	toxic goiter	2836	1.1 (32)	~8
Schmit [16]	(150–400 Gy)	toxic goiter	1357	1.1 (15)	1–13

In radioiodine therapy for non-toxic goiter (adenoma or multinodular goiter), 5% (9/191) of patients developed anti-thyrotropin receptor antibody (TRAb)-positive Graves’ hyperthyroidism after 3 months, and 3% (5/191) developed radiation thyroiditis at 1 month [13]. Positive anti-thyroid peroxidase (TPO) antibodies and higher TRAb levels, albeit within the normal range, at the time of treatment are risk factors, because the incidence of hyperthyroidism was 22% (6/27) in patients with positive anti-TPO antibodies vs 2% (2/103) in those negative for anti-TPO antibodies, and the TRAb levels were higher in hyperthyroid patients than in controls at the time of treatment (3–7 vs 1–3 U/l).

Furthermore, in radioiodine therapy for toxic goiter (adenoma or multinodular goiter), 4% (5/149) of patients shifted from TRAb-negative toxic goiter to TRAb-positive Graves’ hyperthyroidism 3–6 months after treatment [14]. Positivity for anti-TPO antibodies was a risk factor for developing radioiodine-induced hyperthyroidism in this study also.

The incidence of radioiodine-induced Graves’ disease in subjects with thyroid autonomy who were treated with ^131^I (thyroid radiation doses of 150–400 Gy) was reported to be 1.1% (32/2836) with time intervals of ~8 months (range, 4–13 months) [15]. In this study, again, a majority of patients had evidence of pre-existing thyroid autoimmunity, such as a diffuse hypoechoic pattern on thyroid ultrasound, positive anti-TPO autoantibodies, and borderline TRAb titers.

The incidence was also reported to be 1.1% (15/1357) in patients with autonomous thyroid disease between 1 and 13 months after treatment, which increases ~10-fold when anti-TPO antibody levels are elevated before treatment [16].

Overall, it is fairly clear that high-dose irradiation induces thyroid autoimmunity and dysfunction. Thus, the incidences of hypothyroidism, hyperthyroidism and thyroiditis/silent thyroiditis after high-dose external irradiation to the neck regions including the thyroid or as TBI were 2.4% to 31%, 0% to 5% and 0.2% to3%, respectively (Table 1). Similarly, hyperthyroidism can also be induced by high-dose internal irradiation at frequencies ranging from 1% to 5% (Table 2). The studies cited above indicate that a higher dose is a risk for development of thyroid dysfunction. It is easy to understand that a higher dose of radiation (either external or internal) destroys the thyroid gland more efficiently. In the case of the development of hyperthyroidism and thyroiditis, the immune system likely recognizes the thyroid antigens released from the thyroid destroyed by irradiation; so, a higher radiation dose releases more antigens, resulting in a higher chance for induction of thyroid autoimmunity. The existence of thyroid autoimmunity (e.g. positive anti-thyroid (anti-TPO or anti-thyroglobulin) antibodies and/or borderline levels of TRAb) at the time of treatment is another risk factor at least for Graves’ disease induced by high-dose internal irradiation. Thus, it is likely that Graves’ disease may become clinically obvious with ^131^I in the subjects with a propensity to Graves’ disease.

Of further interest, a clear difference exists in the latent periods from the initial diagnosis/treatment to development of hyperthyroidism between external and internal irradiation; years in the former (up to 25 years) and months in the latter (see Tables 1 and 2 for comparison). The reasons for this difference are at present unclear. The existence of a predisposition to thyroid autoimmunity in the cohorts of the internal irradiation study may be a factor in the shorter latency, although this issue was not evaluated in the external irradiation studies.

## THE EFFECT OF MODERATE- TO LOW-DOSE RADIATION

In contrast to high-dose irradiation, the data for the effect of moderate- to low-dose irradiation on thyroid function and autoimmunity are inconsistent.

### External irradiation

A series of follow-up studies on Japanese atomic bomb survivors was mainly conducted by the Radiation Effects Research Foundation (RERF) (Table 3). The initial examinations done in 1974 to 1976 (~30 years after the atomic bombings) [17] demonstrated no increase in the incidence of chronic thyroiditis or hypothyroidism (irrespective of whether autoimmune or non-autoimmune) in the survivors who received >0.1 Gy at the age of <20 years old (*n* = 477). A diagnosis of thyroiditis was made based on the presence of lymphocytic infiltration, positive anti-thyroid antibodies and/or a large goiter.

**Table 3.** The results of studies on thyroid dysfunction and autoimmunity in atomic bomb survivors

Authors [ref.]	Radiation dose or distance from the hypocenter	# subjects	Interval between time of exposure and study (years)	Results in the exposed subjects
Morimoto [17]	>0.1 Gy	477	~30	no increase in the incidence of chronic thyroiditis or hypothyroidism
Ito [18]	<1.5 km	6112	39	higher incidence of hypothyroidism but lower prevalence of anti-microsomal antibodies
Nagasaki [19]	0–>1.0 Gy	2587	39–42	a significant relationship between thyroid radiation dose and antibody^+^ hypothyroidism showing a concave dose response reaching a maximum levels of 0.7 Sv
Fujiwara [20]	0–5.6 Gy	2061	42–44	no increase in anti-thyroid antibodies
Imaizumi [21]	<0.005–>1.0 Gy	4091	55–58	no relationship between thyroid radiation dose and any thyroid dysfunction/autoimmunity
Imaizumi [22]	median 0.182 (range, 0–4.04) Gy	2668	62–66	no relationship between thyroid radiation dose and any thyroid dysfunction/autoimmunity
Yoshimoto [23]	0–>0.5 Gy	3821 autopsy cases	6–40	no increase in the incidence of chronic thyroiditis
Imaizumi [24]	median 0.256 (range, 0.022–1.789) Gy	319 exposed *in utero*	55–58	no relationship between thyroid radiation dose and autoimmune thyroid diseases

On the other hand, the next surveys performed ~40 years after the atomic bombings provided inconsistent results. One study done in 1984 (39 years after the bombings) [18] showed a higher incidence of hypothyroidism in the survivors who were exposed within 1.5 km of the hypocenter (*n* = 6112) as compared with the controls (exposed beyond 3.0 km; *n* = 3047) (1.22% vs 0.35% in males and 7.08% vs 1.18% in females, after adjusting for age), but the prevalence of anti-thyroid microsomal antibodies was unaccountably lower in the former than in the latter (16.4% vs 88.9% in males and 25.3% vs 63.3% in females). Another study (the Nagasaki Adult Health Study, *n* = 2587) conducted from 1984 to 1987 (at 39 to 42 years after the bombings) [19] reported no relationship between the incidence of hypothyroidism/positive anti-thyroid antibodies and thyroid radiation doses (range, 0.39–0.6 Gy), but it found a significant linear–quadratic and concave dose–response relationship in anti-thyroid antibody-positive spontaneous hypothyroidism (autoimmune hypothyroidism), with the maximum prevalence being estimated to be at a dose of ~0.7 Gy. The other study, done in 1987 to 1989 [20], which examined the immunological status of the survivors (whose estimated doses ranged from 0 to 5.6 Gy), found increased prevalence and the titer of rheumatoid factor, but not of other autoantibodies (such as anti-nuclear, anti-thyroglobulin or anti-microsomal antibodies) or immunoglobulin levels in 2061 survivors.

However, the aforementioned significant linear–quadratic and concave dose–response relationship in autoantibody-positive spontaneous hypothyroidism [19] was not replicated, and no relationship between thyroid radiation dose and any pathological thyroid condition was found in the next two reports with longer follow-up periods (55–58 and 62–66 years, respectively, after the atomic bombings) [21, 22].

Thyroid histology was also evaluated in 3821 autopsy cases collected between 1951 and 1985, but no association between radiation exposure and chronic thyroiditis was revealed [23].

Finally, a study of atomic bomb survivors who were exposed *in utero* [24], whose mean maternal uterine radiation dose was 0.256 Gy (range, 0.022–1.789 Gy; *n* = 319), found no association between radiation dose and autoimmune thyroid diseases (such as positive anti-thyroid antibodies, antibody-positive hypothyroidism, antibody-negative hypothyroidism and Graves’ disease).

There is also a report on the Chernobyl nuclear power plant employees who were exposed to a life-time (external) dose of 70–400 mSv (*n* = 71) [25], showing that the absolute values of anti-TPO autoantibodies in this group were slightly, but significantly, higher than the controls (4.5 ± 11.7 IU/ml vs <1.0 ± 2.2 IU/ml, *P* < 0.05), but the proportion of TPO antibody positivity did not differ between the two groups. Serum TSH levels and the prevalence of subjects with elevated TSH levels were significantly higher than in the controls (0.91 ± 0.72 IU/ml vs 0.66 ± 0.47 IU/ml, *P* < 0.05; 7.0% vs 1.7%, *P* < 0.05).

### Internal irradiation

The most serious accident involving internal irradiation was the Chernobyl nuclear power plant accident in 1986, which caused widespread contamination of Russia, Belarus and Ukraine, with released radionuclides, the majority of which were radioactive iodine (Table 4).

**Table 4.** The results of studies on thyroid dysfunction and autoimmunity in Chernobyl

Authors [ref.]	Radiation dose	# subjects	Interval between time of accident and study (years)	Results in the exposed subjects
Ito [26]	various degrees of radioactive contamination	55 054	5–7	an increase in the incidence of chronic thyroiditis
Vykhovanets [27]	^131^I thyroid dose of <1.0–>2.0 mSv	29	7–8	an increase in positivity of anti-thyroglobulin antibodies
Pacini [28]	average Cs of 5.4 Ci/km^2^	287	6–8	an increase in positivity of anti-thyroglobulin/TPO antibodies
Vermiglio [29]	average Cs of 37–185 GBq/km^2^	143	6–8	an increase in positivity of anti-thyroglobulin/TPO antibodies
Pacini [30]	various degrees of radioactive contamination in Belarus	171 thyroid cancer cases	0–10	an increase in chronic thyroiditis and positivity of anti-TPO antibodies
Ivanov [31]	0.132 ± 0.45 (mean + S.D.) Gy		11–13	no significant relationship between radiation dose and the prevalence of chronic thyroiditis
Stezhko [33], Hatch [34], Ostroumova [35] and Tronko [36] (Ukrainian–American Cohort Study of Thyroid Cancer and Other Thyroid Diseases)	average ^131^I thyroid dose of 0.79 (ranged, 0–40.7) Gy	~12 000	12–14	a significant relationship between thyroid dose and the prevalence of hypothyroidism and positivity of anti-thyroid antibodies, but not hyperthyroidism or autoimmune thyroiditis
Agate [32]	various degrees of radioactive contamination	283 in Belarus, 336 in Ukraine and 185 in Russia	13–15	significant increases in the positivity of anti-TPO, not Tg, antibodies in Belarus, and of anti-Tg, not TPO, antibodies in Russia
Ostroumova [37]	average ^131^I thyroid dose of 0.54 (ranged, 0.001–26.6) Gy	10 827	10–17	a significant relationship of thyroid dose with antibody-negative hypothyroidism, but not with antibody-positive hypothyroidism or hyperthyroidism
Kimura [38]	average ^131^I thyroid dose of 0.15–0.65 Gy	300	26–28	no increase in the prevalence of anti-thyroid antibodies or thyroid dysfunction

Several studies were conducted within the first 10 years after the accident. The first study, by Ito *et al.*, who performed a survey 5–7 years after the accident with 55 054 subjects, who were 0–10-year-old children at the time of the accident [26], suggests a possible increase in the incidence of chronic thyroiditis; among the subjects who were found to have ultrasonographic abnormalities and then underwent fine-needle aspiration cytological biopsy, the incidence of chronic thyroiditis was 30% (49/163) in the severely contaminated area vs 14% (4/18) in the relatively less contaminated area. Most of the subjects with chronic thyroiditis (90.2%) were reported to be positive for anti-thyroid antibodies. In the second study, conducted 7–8 years after the accident [27], anti-thyroglobulin antibodies were positive in 81% (25/31) of children with a dose of ^131^I to the thyroid at age 0–7 years at the accident, whereas the percentage for the controls was 17% (7/42); anti-thyroglobulin antibody titer levels were positively correlated with the ^131^I dose to the thyroid. This study also showed higher percentages of CD4^+^ helper T cells, lower percentages of CD8^+^ cytotoxic T cells and increased ratios of CD4^+^/CD8^+^ in children with a thyroid dose of >2 Gy as compared with the controls or those with a thyroid dose of <1 Gy. The third study was done at 6–8 years after the accident with 287 subjects who were 12 years old or less and lived in the area where the average cesium contamination was 5.4 Ci/km^2^ [28]. It showed a higher prevalence of anti-thyroid antibodies, especially anti-TPO antibodies, as compared with the control subjects who lived in the area of iodine-deficiency, with the average Cs contamination being <0.1 Ci/km^2^ (19.5%, 56/287 vs 3.8%, 8/208 for anti-thyroid antibodies, 11.1%, 32/287 vs 0.9%, 2/208 for anti-TPO antibodies). In the fourth study, carried out at the same time period [29], the percentages for positive anti-thyroid antibodies were reported to be 18.9% (27/143; ~4-fold higher) in children at ages of 5–15 years old in the contaminated area (37–185 GBq/km^2^ of ^137^Cs) vs 5% (2/40) in the uncontaminated area (<3.7 GBq/km^2^). Of interest, the incidence seemed to also be higher in subjects who were newborns or *in utero* at the time of the accident in the last two studies. Finally, 51.9% (27/52) and 46.0% (79/171) of patients with thyroid cancers in Belarus who had surgery after the accident showed intrathyroidal lymphocyte infiltration and positive anti-TPO antibodies, respectively, vs 22 9% (22/96) and 23.3% (24/103) in naturally occurring thyroid cancers in Italy and France [30].

In the next 10–20 years after the accident, the first study, undertaken from 1997 to 1999 (at 11–13 years after the accident) [31] showed no significant radiation dose–response of chronic thyroiditis as determined by thyroid ultrasound examination. In the second study, anti-thyroid antibodies as well as thyroid hormones and TSH were measured in sera collected from 1999 to 2001 (at 13 to 15 years after the accident) [32]. The prevalence of anti-TPO autoantibodies was still higher in contaminated areas than in non-contaminated areas (6.4%, 18/283 vs 2.4%, 6/258), but it was lower than that determined 6–8 years after the accident (11.1%) [28].

In a series of the Ukrainian–American Cohort Study of Thyroid Cancer and Other Thyroid Diseases Following the Chornobyl Accident [carried out 12–14 years after the accident (1998–2000), in which the mean thyroid ^131^I dose was 0.79 Gy, range, 0–40.7 Gy [33], a significant correlation between thyroid ^131^I dose and the prevalence of hypothyroidism (6.1%, 719/11 853) and thyroid autoimmunity (i.e. positive anti-TPO antibodies) (11.9%, 1456/12 240), but not the prevalence of hyperthyroidism (0.6%, 76/11 853) or autoimmune thyroiditis (1.6%, 196/12 240), was observed [34–36]. The risk of hypothyroidism was higher in the individuals with anti-TPO antibodies of >60 U/ml than in those with anti-TPO-antibodies of <60 U/ml (8.5%, 128/1513 vs 5.3%, 591/11 059) [35]. Similar data were also reported in the other study done in 1996–2003 [37]. Of interest, in one of these studies [36], done between 12 to 14 years after the accident, the linear-exponential model showed the concave relationship between thyroid 131I dose and the prevalence of anti-TPO antibodies, as also shown in the study of atomic bomb survivors [19].

However, the most recent study performed in 2012–2014 (26–28 years after the accident) with 300 subjects (who were at ages of 0–5 years at the time of the accident) no longer showed any difference in the prevalence of anti-thyroid antibodies (21%, 63/300 vs 17.3%, 52/300, *P* > 0.05) or subclinical/clinical thyroid dysfunction (3%, 10/300 vs 1%, 3/300, *P* > 0.05) between the contaminated and non-contaminated areas [38].

Studies were also performed at different time points in a Marshallese population, where people were exposed to radioactive fallout from the nuclear weapons test in 1954 (Table 5). Larsen *et al.* [39] performed their examinations ~20 years later and found an increased prevalence of anti-thyroid antibody-negative hypothyroidism (16.3%, 14/86) in exposed (estimated thyroid doses ranged from 1.35 to 21 Gy) vs <1% in unexposed people. The majority of people who developed hypothyroidism were infants or *in utero* at the time of exposure, and dysfunction occurred about 10 years after exposure. In contrast, however, Takahashi *et al.* [40] found no increase in the prevalence of autoimmune thyroiditis (1.1%, 34/3000; diagnosed by ultrasonographic findings or positive anti-thyroid antibodies) or hypothyroidism (1.5–3% in different regions) in their investigation, carried out 1993–1997, i.e. 39–43 years later. However, radiation doses were not described in that report.

**Table 5.** The results of studies on thyroid dysfunction and autoimmunity in the Marshall Islands and nuclear sites

Authors [ref.]	Place	Radiation dose	# subjects	Interval between time of accident and study (years)	Results in the exposed subjects
Larsen [39]	Marshallese islands	1.35–21 Gy	86	~20	an increase in the prevalence of anti-thyroid antibody-negative hypothyroidism
Takahashi [40]		unknown	3000	39–43	no increase in autoimmune thyroiditis or hypothyroidism
Lyon [41]	Nevada nuclear weapons test site	120 ± 167 mGy	2473	~35	an increased risk for autoimmune thyroid disease in the highest dose exposure group (>400 mGy)
Davis [42]	Hanford nuclear site	mean of 174 (range, 0.003–2823) mGy	3440	~40	no significant relationship between radiation dose and thyroid dysfunction/autoimmunity
Mushacheva [43]	Mayak nuclear weapons facility	thyroid radiation dose of up to 8 Gy	581	~50	no increase in thyroid dysfunction/autoimmunity

There are some further publications reporting the health consequences of ^131^I exposure. The long-term follow-up study of children exposed to radioactive iodine at the Nevada nuclear weapons test site from 1951 to 1962 (carried out from 1985 to 1986; ~35 years after exposure) (mean dose, 120 ± 167 mGy) found an increased risk of autoimmune thyroid disease (diagnosed by positive anti-thyroglobulin or microsomal antibodies) for those in the highest dose exposure group (>400 mGy) [41]. In contrast, the retrospective study of atmospheric release of ^131^I from the Hanford nuclear site from 1944 to 1957 (carried out from 1992 to 1997; ~40 years after exposure) found that there was no significant correlation between radiation dose and thyroid dysfunction or autoimmunity, including autoimmune thyroiditis diagnosed by positive anti-TPO, microsomal or thyroglobulin autoantibodies, hypothyroidism or autoimmune thyroiditis, with hypothyroidism diagnosed by very high titers of autoantibodies in subjects who received estimated radiation doses of 0.003–2823 mGy (median, 97 mGy; mean, 174 mGy) [42]. Furthermore, people who were born in 1952 or 1953 and exposed to radionuclides (mostly ^131^I) released from the Mayak nuclear weapons facility site from 1948 to 1960 also did not show any increase in the prevalence of autoimmune thyroid disease (diagnosed by the presence of hypothyroidism, positive anti-TPO antibodies, or findings of ultrasound examination or palpitation) (6.4%, 37/581 for exposed vs 8.0%, 25/313 for non-exposed) when evaluated ~50 years later [43].

**Fig. 1.**
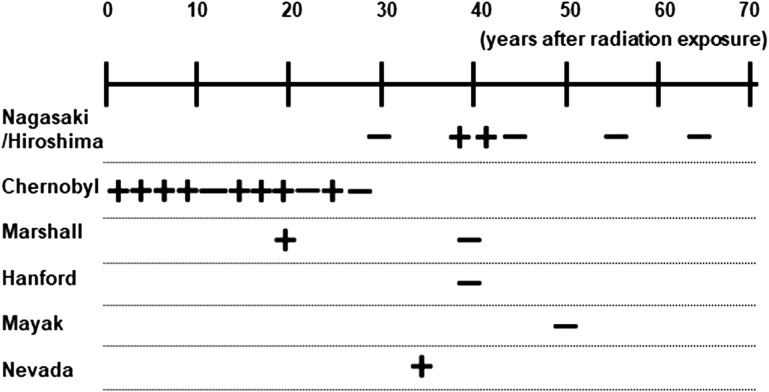
Summary of studies on the consequences of exposure to moderate to low-dose irradiation on thyroid dysfunction/autoimmunity. ‘+’ indicates the articles reporting increase(s) in the incidence of thyroid dysfunction/autoimmunity, and ‘−’ indicates the articles reporting no increase in the incidence of thyroid dysfunction/autoimmunity. Multiple studies were done on Nagasaki/Hiroshima atomic bomb survivors, and inhabitants in/around Chernobyl and the Marshall Islands.

Most of the studies on the effects of moderate- to low-dose irradiation on thyroid function/autoimmunity are summarized in Tables 3 to 5. In contrast to high-dose irradiation, the data vary widely in the moderate- to low-dose irradiation studies. One should be cautious when interpreting these data, because the results of these types of epidemiological studies can be influenced by numerous factors, such as different iodine status [44], different pattern of radiation exposure (external vs internal irradiation; acute vs chronic exposure, etc.), a lack of consensus on the criteria for thyroid autoimmunity/autoimmune thyroid disease, inaccuracy of radiation dose calculation/estimation and limited sample sizes. Thus, it is difficult to a draw the definitive conclusion from these data. Nevertheless, as proposed by others previously [32, 45], the studies done at different time points after exposure in Japanese atomic bomb survivors and the residents around Chernobyl and in the Marshall Islands may point to a trend suggesting that the effect of moderate- to low-dose irradiation on thyroid autoimmunity/dysfunction is transient (see Fig. 1), without irreversible development of autoimmunity/dysfunction. However, this interpretation also requires caution, because time windows for the appearance of thyroid autoimmunity/dysfunction are different in these three cohorts: at ~40 years after atomic bombing, during the first 20 years after the Chernobyl accident, and at ~20 years after fallout in the Marshall Islands.

## CONCLUSION

Taken together, it is fairly clear that high-dose irradiation, whether external or internal, is strongly associated with a risk of hypothyroidism (with the prevalence ranging from 2.4% to 31%) and of Graves’-like hyperthyroidism (with the prevalence being up to 5%). It is possible that high-dose irradiation–induced hypothyroidism may occur stochastically with some frequency, because high-dose irradiation destroys the thyroid gland by inducing tissue damage, including damage to the vessels and parenchymal cells [46]. On the other hand, however, hyperthyroidism may be induced by recognition of autoantigens released from a damaged thyroid gland by the immune system, but only in subjects who are immunologically prone to developing Graves’ disease.

By contrast, the effects of moderate- to low-dose irradiation on thyroid autoimmunity and dysfunction, particularly on hypothyroidism and anti-thyroid antibodies, are inconsistent. This is at least in part attributed to the facts that the epidemiological data cited here were likely influenced by the numerous factors mentioned before. Thus, a definitive conclusion cannot be drawn at present. However, some data may suggest a transient effect of moderate- to low-dose irradiation on hypothyroidism and autoimmune thyroiditis, implying that the effect, if it exists, is reversible. There have so far been no reports showing a significant increase in the prevalence of Graves’ hyperthyroidism due to moderate- to low-dose irradiation. There was a suggestion of a significant correlation between thyroid dose and the relative risk of Graves’ disease in atomic bomb survivors (*P* = 0.1, in ref. [21]), which was however not replicated in a more recent study [22]. Although the recent report from the ICRP in 2012 [47] supports our conclusions in this review, further studies will be necessary to elucidate the effect of moderate- to low-dose irradiation on thyroid autoimmunity/dysfunction.
